# Stability of Phenyl-Modified Triphenylphosphonium Conjugates and Interactions with DTPA

**DOI:** 10.1021/acsomega.2c06525

**Published:** 2022-12-13

**Authors:** Hannah K. Gruenwald, Robert J. Kerns

**Affiliations:** University of Iowa, 438 College of Pharmacy Building (CPB), 180 S. Grand Ave., Iowa City, Iowa 52242, United States

## Abstract

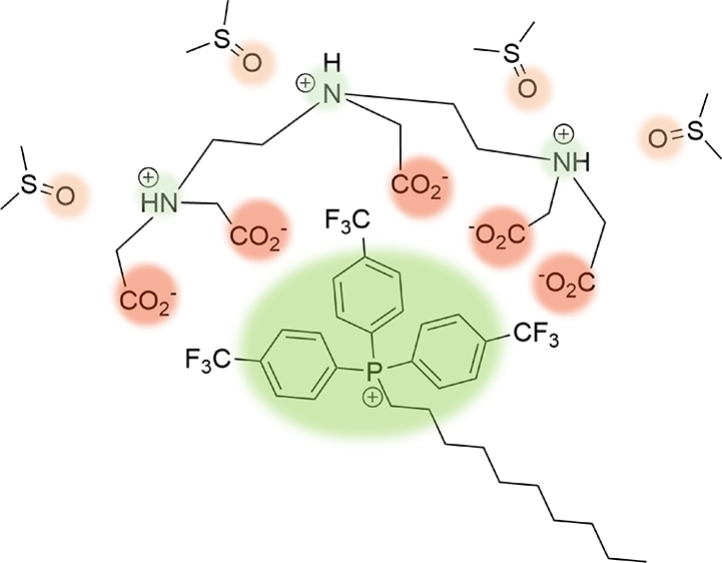

Triphenylphosphonium (TPP^+^) conjugates are effective in targeting drugs and probes to the mitochondria due to their lipophilic character that allows them to readily cross membranes and their large cationic radius that enables mitochondrial uptake because of the mitochondria’s negative membrane potential. TPP^+^ conjugates, while effectively sequestered by the mitochondria, are also known to uncouple oxidative phosphorylation (OXPHOS) and depolarize the mitochondrial membrane. xTPP^+^ conjugates with para-substitutions of functional groups on the phenyl rings of the TPP^+^ moiety display different levels of dose-mediated cytotoxicity due to differing potencies of uncoupling. xTPP^+^ conjugates having a para CF_3_ group substituted on the phenyl rings have been shown to afford significantly reduced uncoupling potency. In the present study, the analysis of a CF_3_-TPP^+^ conjugate with a decyl linker for stability revealed instability specific to the presence of DMSO in aqueous alkaline buffer. It is also demonstrated that the metal chelator, DTPA, forms a noncovalent protective complex with TPP^+^ moieties and prevents degradation of the CF_3_-TPP^+^ conjugate in aqueous DMSO. The stability of different xTPP^+^ conjugates and their interactions with DTPA are reported.

## 1 Introduction

### 1.1 Triphenylphosphonium Targeting of Mitochondria

The mitochondrion is a target for various drugs and probes as it plays a role in various cellular functions including calcium homeostasis, redox-cell signaling, ATP synthesis, and apoptosis. The dysfunction of mitochondria leads to disease states including neurodegenerative and metabolic diseases, aging, and cancer.^1,2^ The triphenylphosphonium moiety (TPP^+^) is a diffused lipophilic cation that is sequestered by mitochondria due to its large cationic radius and the negative mitochondrial membrane potential. By linkage to a TPP^+^ moiety, small molecules can be delivered to the mitochondria with up to a 1000-fold higher accumulation relative to the outside of the cell.^3^ TPP^+^ conjugates have therefore been utilized in research and medicine as an effective way to deliver cargo to the mitochondria.^4^

**Figure 1:**
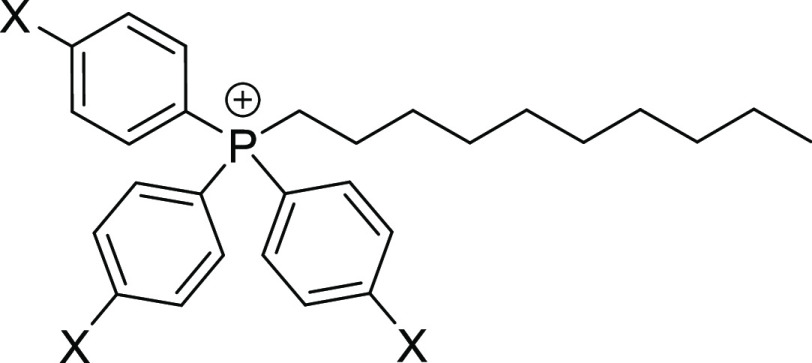
Structure of para-substituted triphenylphosphonium conjugates (xTPP^+^) with a decyl (DC) linker used in this study. In parent xTPP^+^-DC, X = H; in other conjugates, X = CF_3_, F, Cl, CH_3_, and OMe.

While effective in mitochondrial targeting, TPP^+^ conjugates have demonstrated detrimental effects on mitochondrial bioenergetics by causing proton leak and uncoupling of oxidative phosphorylation (OXPHOS), thereby decreasing ATP production.^5^ This led to the development of phenyl-substituted xTPP^+^ derivatives, as shown in Figure 1, that separated mitochondrial uptake from uncoupling potency and cytotoxicity.^3^ Specifically, conjugates with electron-withdrawing or electron-donating substituents at the para position of the TPP^+^ phenyl rings display reduced uncoupling or increased uncoupling, respectively, while maintaining mitochondrial accumulation.^6,7^ Trifluoromethylphenyl phosphonium (CF_3_-TPP^+^) is a promising TPP^+^ derivative to replace the parent TPP^+^ group in drug and probe conjugates because of its comparatively lower cytotoxicity. Conjugates of the CF_3_-TPP^+^ group have been shown to have significantly reduced uncoupling effects as compared to conjugates comprised of the unsubstituted TPP^+^, with no increase in proton leak as a consequence of uncoupling OXPHOS.^3^

### 1.2 Potential Instability of New xTPP^+^ Conjugates

While para-substituted xTPP^+^ derivatives have shown no instability with long-term storage and use, we observed the disappearance of a unique CF_3_-TPP^+^ conjugate in aqueous dimethyl sulfoxide (DMSO) stock solutions. This observation led to concerns regarding the stability, and potential instability, of different xTPP^+^ conjugates in experimentally relevant solutions and biologically relevant conditions. TPP^+^ conjugates undergo several known reactions, including hydrolysis under strongly basic conditions, to give the phosphine oxide product.^8^ There are also several known reactions in organic chemistry where DMSO acts as a nucleophile in oxidation chemistry.^9,10^ As such, we set out to further investigate if DMSO and/or pH was playing a role in the potential degradation of CF_3_-TPP^+^ conjugates specifically and to evaluate the stability of different xTPP^+^ groups more broadly in general.

## 2 Results and Discussion

### 2.1 Stability of CF_3_-TPP^+^-DC in Buffer Solutions

With a CF_3_-TPP^+^ conjugate being the xTPP^+^ derivative to present stability concerns, the stability of CF_3_-TPP^+^-DC (Figure 1) was assessed first. Due to the high lipophilic character of xTPP^+^-DC conjugates, organic cosolvents are necessary to dissolve the conjugates in aqueous buffer. Experimental conditions were chosen to be consistent with conditions used when handling these and preparing stock solutions in buffers. The mitochondrial pH ranges from 7.4 to 8.3; therefore, stabilities in alkaline aqueous buffers with cosolvents were assessed, in addition to neutral and acidic pH.^11^

**Figure 2:**
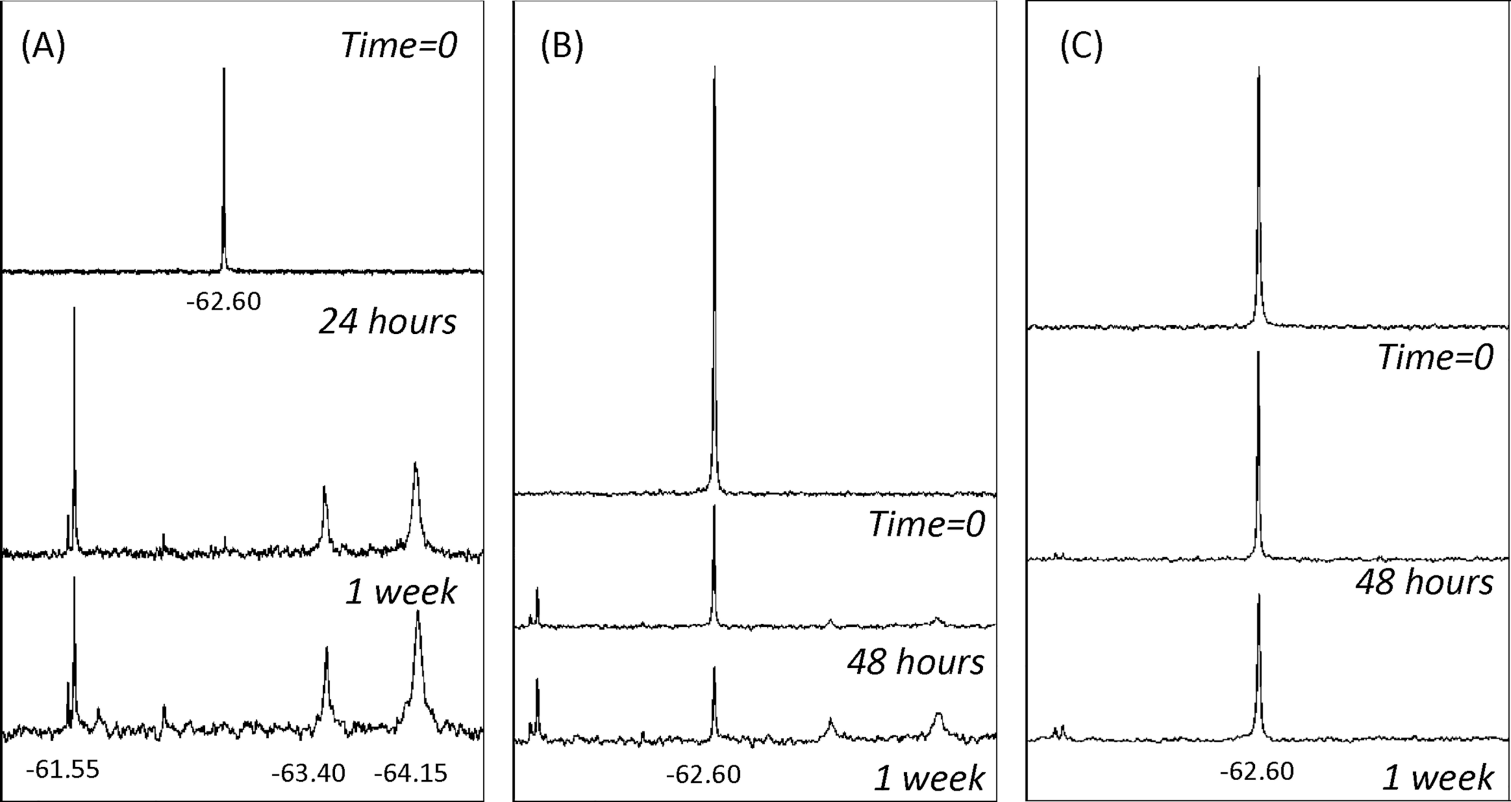
Degradation of CF_3_-TPP^+^-DC as followed by ^19^F NMR: (A) 1:1 DMSO-*d*:Tris buffer, pH 8.3; (B) 1:1 DMSO-*d*:Tris buffer, pH 7.8; (C) 1:1 DMSO-*d*:Tris buffer, pH 7.4.

Cosolvents used with xTPP^+^ conjugates have included DMSO, ethanol, and acetonitrile. Therefore, deuterated versions of each solvent were used to make stock solutions in buffers, which allowed for NMR analysis. As an initial control, CF_3_-TPP^+^-DC was dissolved in 100% DMSO-*d*, ACN-*d*, EtOD, and CDCl_3_ and analyzed by ^1^H and ^19^F NMR. Degradation was not observed in any of these solvents over 2 weeks. Having demonstrated stability in a pure organic solvent, CF_3_-TPP^+^-DC was then dissolved in a deuterated solvent and diluted with Tris buffer at pH 7.4, 7.8, and 8.3, to give a 1:1 cosolvent-to-buffer ratio at 1 mg/mL CF_3_-TPP^+^-DC. This concentration of CF_3_-TPP^+^-DC enabled the obtention of high-quality NMR spectra with a limited number of scans. A 50% organic solvent is required to solubilize CF_3_-TPP^+^-DC at this concentration. Of the buffer and cosolvent combinations tested, degradation of CF_3_-TPP^+^-DC was only observed in 1:1 DMSO-*d*:Tris buffer at pH 7.4, 7.8, and 8.3. No degradation was observed at pH 2. As shown in Figure 2, the rate of degradation is pH-dependent. In 1:1 DMSO:Tris at pH 8.3, the ^19^F NMR signal for CF_3_-TPP^+^-DC (−62.60 ppm) was almost completely indiscernible after only 24 h due to significant degradation (Figure 2A). At pH 7.8, degradation was slower but observable after 48 h (Figure 2B). Degradation at pH 7.4 was minimal at 1 week (Figure 2C). No degradation was observed under identical conditions when EtOD-*d* or ACN-*d* was used as an aqueous cosolvent in place of DMSO-*d*.

**Figure 3:**
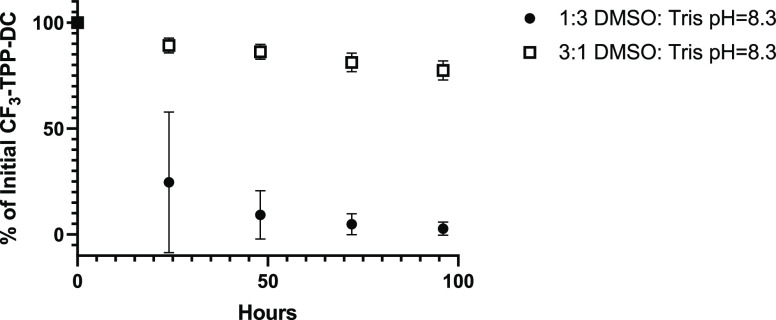
Rate of degradation of CF_3_-TPP^+^-DC at different ratios of DMSO-*d* to Tris buffer at pH 8.3. Degradation is represented as the percent of initial CF_3_-TPP^+^-DC concentration over 96 h in 0.2 mg/mL solutions of 1:3 DMSO:Tris at pH 8.3 (closed circles) and 3:1 DMSO:Tris at pH 8.3 (open squares).

Having identified DMSO as uniquely contributing to the instability of CF_3_-TPP^+^-DC in alkaline buffer, the DMSO–buffer ratio was varied to investigate the influence of DMSO concentration on degradation. At 0.2 mg/mL, CF_3_-TPP^+^-DC was dissolved in 3:1 or 1:3 DMSO:Tris at pH 8.3, and degradation was followed by analytical HPLC. As shown in Figure 3, the degradation rate decreased with increased concentration of DMSO, and prior control studies demonstrated no degradation of CF_3_-TPP^+^-DC in 100% DMSO-*d*. CF_3_-TPP^+^ conjugated with an ethyl chain in place of the decyl chain also showed degradation in DMSO:Tris at pH 8.3 (data not shown), further supporting a general trend of the CF_3_-TPP^+^ moiety possessing a unique reactivity in alkaline aqueous DMSO solutions.

**Figure 4:**
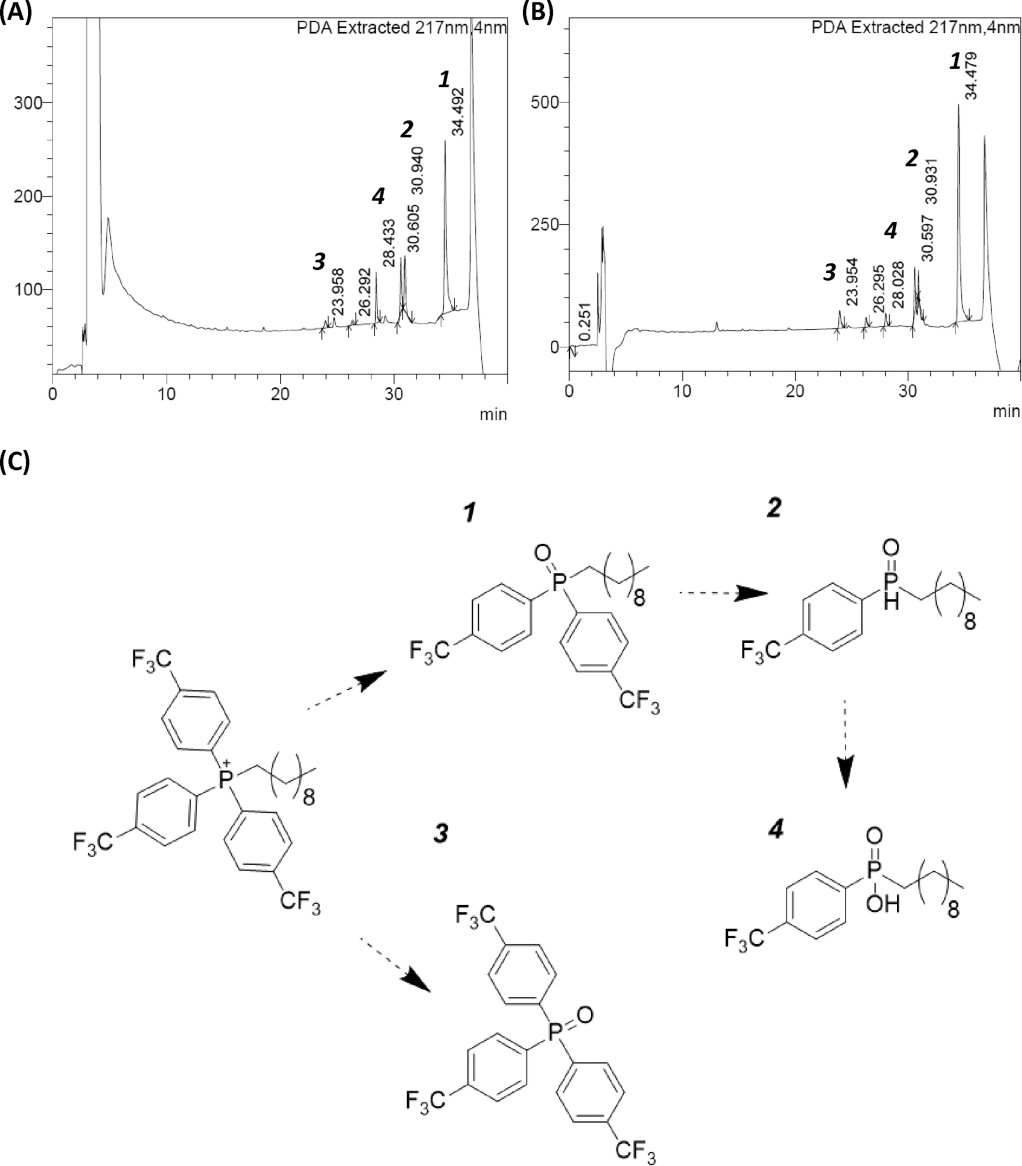
Hydrolysis of CF_3_-TPP^+^-DC with DMSO in alkaline buffer or aqueous NaOH. (A, B) Comparison of CF_3_-TPP^+^-DC degradation seen in (A) 1:1 DMSO:Tris buffer at pH 8.3 and (B) 1:1 1 N NaOH:acetonitrile by analytical HPLC. Common peaks are labeled in the order they appeared (1–4). Unlabeled common peaks were not stable enough to be isolated for further analysis. (C) Proposed hydrolysis degradation pathway of CF_3_-TPP^+^-DC from degradation products.

Tris buffer is somewhat unique because the Tris molecule contains both primary hydroxyl and primary amine groups. To elucidate any potential role of Tris itself in the degradation of CF_3_-TPP^+^-DC, an additional experiment was conducted using HEPES buffer at pH 7.4 and 8.3 with the DMSO cosolvent. Degradation of CF_3_-TPP^+^-DC in 1:1 alkaline HEPES buffer:DMSO-*d* gave identical degradation peaks by ^19^F NMR, albeit at comparatively slower rates. To assess whether pH plays a specific role in the degradation of CF_3_-TPP^+^-DC, an experiment was conducted using 1:1 DMSO:Tris at an acidic pH of 2.0. No degradation was observed in this condition. These results suggest that degradation is dependent on the presence of DMSO in alkaline aqueous buffer and is not specific to Tris buffer. To compare the degradation of CF_3_-TPP^+^-DC in DMSO and alkaline basic buffer with hydrolysis by NaOH, CF_3_-TPP^+^-DC was treated with 1:1 1 N NaOH:acetonitrile at 1 mg/mL, and solutions were analyzed using analytical HPLC. While we had initially discounted degradation by hydrolysis due to the apparent necessary role of DMSO, the pattern of degradation products seen with NaOH (Figure 4B) is the same pattern of products observed with aqueous DMSO at pH > 7.4 (Figure 4A), suggesting that DMSO-mediated hydrolysis is the mechanism of degradation for these conjugates in alkaline buffer in the presence of DMSO.

### 2.2 Characterization of Products from the Hydrolysis of CF_3_-TPP^+^-DC in DMSO and Alkaline Buffer

To elucidate the identity of degradation products formed from the hydrolysis of CF_3_-TPP^+^-DC, semipreparative HPLC was used to isolate compounds that correlated to product peaks observed by analytical HPLC (Figure 4). Separated compounds from semiprep HPLC were analyzed by mass spectrometry, and those stable enough to obtain mass (peaks 1–4) are shown in Figure 4. As expected, phosphine oxides (1) and (3) (Figure 4C) are formed from the loss of CF_3_-toluene or the decyl chain, respectively. This further supports that degradation is consistent with hydrolysis. It is proposed that the hydrolysis of CF_3_-TPP^+^-DC in buffer at pH > 7 occurs in the presence of DMSO because DMSO is likely acting as a nucleophile and attacking the phosphorus cation, catalyzing or initiating hydrolysis to the phosphine oxide (1). The loss of an additional phenyl ring affords the phenyl phosphine oxide (2). Product (4) was characterized by the loss of two phenyl rings and the addition of two oxygen molecules by mass. While a phosphine ester or dioxirane would also correspond to the identified mass and could be anticipated products under oxidative conditions, the acid product is proposed as it has been reported to arise from phosphine oxides.^12,13^

### 2.3 Analysis of the Potential Role of Metals in CF_3_-TPP^+^-DC Degradation

In a set of parallel studies to the hydrolysis studies above, we had set out to test for the potential role of metals contributing to an oxidative mechanism of CF_3_-TPP^+^-DC degradation. Because DMSO plays a role in some oxidation reactions, and metals often contribute to redox reactivity, we set out to test for these interactions by using metal chelators to sequester metals in CF_3_-TPP^+^-DC solutions.^9,10^ Metal chelators DTPA, EDTA, and Chelex100 were used to sequester multivalent cations that are commonly involved in redox chemistry. First, EDTA was added to 1:1 DMSO:Tris buffer at pH 8.3 to give EDTA:CF_3_-TPP^+^-DC at a 1:1 molar ratio. Incubation for 1 week showed that degradation proceeded at the same rate as the control without EDTA present, suggesting that metals do not play a role in degradation. However, addition of EDTA would not remove metals, only complex with metals in solution, so an additional experiment using Chelex100 to pull metals out of the solution entirely was conducted to confirm that metals do not play a role in degradation. In this second experiment, DMSO and Tris buffer at pH 8.3 with CF_3_-TPP^+^-DC were incubated with Chelex100 overnight. Perhaps to be expected, CF_3_-TPP^+^-DC was removed from solution by the Chelex100 resin, as shown by the analytical HPLC analysis (data not shown). In a third experiment, DTPA was added to a solution of 1:1 DMSO:Tris buffer at pH 8.3 to give a 1:1 molar ratio with CF_3_-TPP^+^-DC. Unlike EDTA, DTPA completely inhibited the degradation of CF_3_-TPP^+^-DC. These results suggested that either DTPA was interacting with CF_3_-TPP^+^ to block hydrolysis to the phosphine oxide or that DTPA, but not EDTA, sequesters a metal involved in the mechanism of degradation. To fully remove metals in a final experiment, all buffers and cosolvents were preincubated with Chelex100 overnight and filtered to remove Chelex100 before addition of CF_3_-TPP^+^-DC. Hydrolysis in DMSO:Tris buffer proceeded at a normal rate, suggesting no role for metals in the hydrolysis of CF_3_-TPP^+^-DC.

### 2.4 xTPP^+^ Interactions with Metal Chelators

**Figure 5:**
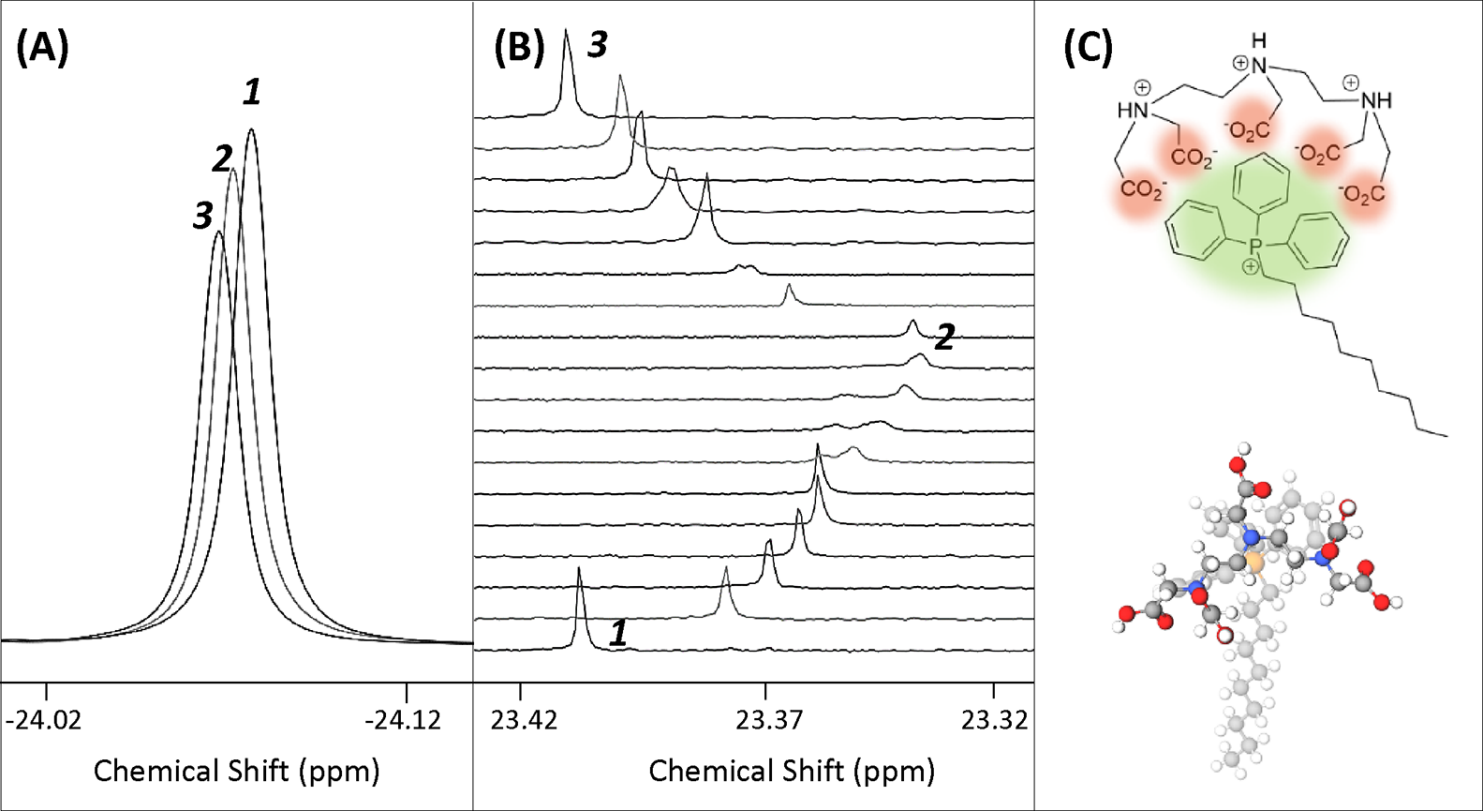
Binding interaction of DTPA with xTPP^+^ conjugates as demonstrated by NMR. (A) ^19^F spectra demonstrating a change in the electronic environment of CF_3_-TPP^+^-DC fluorine nuclei upon addition of DTPA to solution of 1:1 ACN-*d*:Tris buffer at pH 7.4 and 1 mg/mL CF_3_-TPP^+^-DC. (1) No DTPA present; (2) 1:1 molar ratio of DTPA to CF_3_-TPP^+^-DC; (3) 2:1 molar ratio of DTPA to CF_3_-TPP^+^-DC. (B) Stacked ^31^P NMR spectra of TPP^+^-DC demonstrating the strength of interaction between TPP^+^-DC and DTPA at varied molar ratios indicated by a chemical shift change. DTPA was titrated into a solution of 1:1 ACN-*d*:Tris buffer at pH 7.4 and 1 mg/mL TPP^+^-DC. (1) No DTPA present; (2) 1:1 molar ratio of DTPA to TPP^+^-DC; (3) 2:1 molar ratio of DTPA to TPP^+^-DC. The spectra with two peaks are due to insufficient time for the solution to reach equilibrium. (C) 2D and 3D representations of the suggested complex between the triphenylphosphonium cation and the metal chelator DTPA.

The above experiments demonstrated that DTPA in solution, but not EDTA, prevented the DMSO-mediated hydrolysis of CF_3_-TPP^+^-DC. We hypothesized that DTPA, specifically, was interacting with the CF_3_-TPP^+^ moiety, therefore blocking DMSO-mediated hydrolysis. To assess this potential interaction, ^19^F NMR and ^31^P NMR were used to analyze solutions of CF_3_-TPP^+^-DC in 1:1 ACN-*d*:Tris at pH 7.4 with (1) no DTPA, (2) 1:1 DTPA:CF_3_-TPP^+^-DC, and (3) 2:1 DTPA:CF_3_-TPP^+^-DC. While no chemical shift change was observed upon addition of DTPA by ^31^P NMR, an observable change in signal was seen by ^19^F NMR (Figure 5A). The 2:1 DTPA-to-CF_3_-TPP^+^-DC sample showed a larger chemical shift change than the 1:1 molar ratio sample, indicating an increasing change in the electronic environment of the fluorine nuclei upon addition of DTPA to solution. This suggested an interaction between anionic DTPA and the diffuse cationic ring system of CF_3_-TPP^+^-DC (Figure 5C). We hypothesize that the electron-withdrawing character of the CF_3_ substituent prevents the electronic interaction from affecting the central phosphorus atom; therefore, no chemical shift change is seen by ^31^P NMR. A chemical shift change is seen by ^19^F NMR, however, due to the fluorine molecules’ proximity to the carboxylate anions.

Upon demonstrating that an interaction occurs between DTPA and CF_3_-TPP^+^-DC, it was important to determine if DTPA complexes with the parent TPP^+^ group. Having no fluorine substituents on parent TPP^+^-DC, ^31^P NMR was used to analyze interactions. A large change in chemical shift in the ^31^P NMR spectra was observed for TPP^+^-DC as DTPA was titrated into TPP^+^-DC in 1:1 ACN-*d*:Tris at pH 7.4 (Figure 5B). The chemical shift change was the most drastic at a 1:1 molar ratio between TPP^+^-DC and DTPA, suggesting the strongest interaction at a 1:1 ratio. Continuing to titrate DTPA into the solution to reach a 2:1 molar ratio with excess DTPA results in the chemical shift returning to its original frequency. It is hypothesized that electrons from the DTPA carboxylate anions are donated into the TPP^+^ ring system and reach the central phosphorus through resonance effects, increasing the shielding felt by this nucleus and causing a chemical shift change in ^31^P NMR. While the outcome of the electron density on the central phosphorus atom differs between CF_3_-TPP^+^-DC and TPP^+^-DC, the encapsulation of the diffused cationic moiety of xTPP^+^ is proposed to be similar, as shown in Figure 5C.

The interaction of TPP^+^ with DTPA is hypothesized to protect CF_3_-TPP^+^-DC from degradation in alkaline buffer and DMSO stock solutions. It also raises concern regarding the use of DTPA, and possibly EDTA and other metal chelators, in experiments with xTPP^+^ conjugates. Attempts to obtain the crystal structure of a TPP^+^ conjugate with DTPA have not been successful to date.

### 2.5 CF_3_-TPP^+^-DC Stability under Other Biologically Relevant Conditions

Experiments assessing the stability of CF_3_-TPP^+^-DC in various experimentally relevant conditions confirmed the general stability of this xTPP^+^ derivative. Solvent and cosolvent conditions caused no degradation, except for hydrolysis with aqueous DMSO at pH > 7. To generate a more comprehensive understanding of the stability of CF_3_-TPP^+^-DC, its reactivity in other relevant conditions was probed. The potential role of ionic strength on the hydrolysis of CF_3_-TPP^+^-DC was evaluated by adding NaCl or KCl up to 278 mM in solutions of CF_3_-TPP^+^-DC in neutral aqueous DMSO. No degradation was observed, indicating that ionic strength does not contribute to degradation of CF^3^-TPP^+^-DC. To elucidate the potential role of light and oxygen in the degradation of CF_3_-TPP^+^-DC, samples in Tris at pH 8.3 and DMSO were prepared and stored either under argon or in the dark. In either condition, degradation was still observed, suggesting that neither light nor oxygen played an essential role in the degradation of CF_3_-TPP^+^-DC.

As reactive oxygen species (ROS) are prevalent in the mitochondria, solutions with hydrogen peroxide (H_2_O_2_) in aqueous ACN were prepared to assess the potential role of ROS in degradation in CF_3_-TPP^+^-DC, as well as TPP^+^-DC and OMe-TPP^+^-DC (Figure 1). We found OMe-TPP^+^-DC as an important derivative to include in these studies as methoxy radicals have reportedly been formed in the presence of peroxide, potentially affording a different mechanism of reactivity with H_2_O_2_ than other xTPP^+^ derivatives.^14,15^ The xTPP^+^-DC conjugates were dissolved in solutions of two different concentrations of H_2_O_2_. To solutions of xTPP^+^-DC conjugates were added (1) the mitochondrial concentration of H_2_O_2_ (5 × 10^–9^ M) and (2) 2 × 10^7^ times the mitochondrial concentration of H_2_O_2_ (0.1 M) every 45 min for 4.5 h. No degradation was seen with any of the three xTPP^+^-DC conjugates.^14^ Under the hypothesis that DMSO acts as a nucleophile that initiates the conversion of CF_3_-TPP^+^-DC to the phosphine oxide, the stability of this xTPP^+^ conjugate was analyzed in the presence of *N*-acetyl-l-cysteine, a relevant nucleophile, because of the prevalence of thiol residues in mitochondria and cells.^16^ At 1 mg/mL of each CF_3_-TPP^+^-DC and *N*-acetyl-l-cysteine in 1:1 ACN:Tris at pH 8.3, no reaction was seen over the course of 2 weeks. Of all conditions tested, degradation was not observed for CF_3_-TPP^+^-DC except when in the presence of both DMSO and alkaline buffer.

### 2.6 Stability of Other xTPP^+^ Derivatives in DMSO and Alkaline Buffer

Having established the specific conditions that cause the hydrolysis of CF_3_-TPP^+^-DC, we next assessed the stability of members of a previously reported panel of xTPP^+^ conjugates (Figure 1) in 1:1 DMSO:Tris buffer at pH 8.3 and 1:1 ACN:Tris buffer at pH 8.3 as a control. All xTPP^+^-DC conjugates, excluding CF_3_-TPP^+^-DC, were stable under both buffer conditions, as followed over 2 weeks by analytical HPLC. These results indicate that the hydrolysis of xTPP^+^-DC conjugates in alkaline aqueous DMSO is specific to the CF_3_ conjugate. It is hypothesized that the instability of the CF_3_-TPP^+^-DC conjugate is because of the highly electron-withdrawing character of the CF_3_ substituent weakening the phosphorus–carbon bond. It is expected that addition of other strongly electron-withdrawing groups to the aryl rings of xTPP^+^ conjugates will similarly have some instability in alkaline aqueous DMSO solutions.

## 3 Conclusions

Findings demonstrate that the CF_3_-TPP^+^ group is stable in most biologically relevant and experimental conditions but undergoes hydrolysis in alkaline aqueous DMSO. All other members of the xTPP^+^ panel (Figure 1) demonstrate stability under this condition. Metal chelator studies suggest that metals do not play a role in the degradation of CF_3_-TPP^+^-DC and revealed the formation of a protective complex between CF_3_-TPP^+^-DC and DTPA. Initially, hydrolysis was discounted as the degradation mechanism of CF_3_-TPP^+^-DC due to the seemingly necessary role of DMSO, and a solvent-specific, DMSO-mediated oxidation mechanism was assumed. However, upon comparing HPLC data of degradation in DMSO and alkaline buffer to the degradation seen by NaOH and acetonitrile, identical degradation peaks are observed. While other mechanisms may play a role, this suggests that DMSO is sensitizing CF_3_-TPP^+^ conjugates to hydrolytic reactions. Because of this observation, DMSO should not be used as a cosolvent to prepare solutions of CF_3_-TPP^+^ conjugates at pH > 7.

Experiments with DTPA and Chelex100 indicate that metal chelators for multivalent cations should be used with caution in studies employing TPP^+^ conjugates, and potentially studies using diffused lipophilic cations, in general. xTPP^+^ conjugates interact with DTPA and Chelex100 resin, and CF_3_-TPP^+^-DC is protected from hydrolysis in DMSO and alkaline buffer when DTPA is present. It is hypothesized that a noncovalent complex occurs between the anionic carboxylate groups of DTPA with the diffused lipophilic cation of both xTPP^+^ conjugates, which leads to further questions about the nature of the DTPA interaction with lipophilic cations, in general. We did not see protection of degradation of CF_3_-TPP^+^-DC in the presence of EDTA, which might ion-pair poorly, while DTPA is larger and is therefore able to more fully encapsulate the TPP^+^ cation.

Over the years, many research groups have employed TPP conjugates, including those dedicated to developing the methodology.^17−19^ The results of the study here indicate that in general, xTPP^+^ derivatives are as stable as the parent TPP^+^. However, there are specific conditions, including alkaline buffer with DMSO cosolvent, that should be avoided when working with CF_3_-TPP^+^ conjugates and likely any other triphenylphosphonium conjugates where the aromatic rings are substituted with highly electron-withdrawing groups. Metal chelators such as DTPA should also be used with caution with xTPP^+^ conjugates, and appropriate controls performed if used, due to their noncovalent interactions with the diffused cationic center of the TPP^+^ moiety.

## 4 Experimental Procedures

### 4.1 Synthesis

xTPP^+^ conjugates were synthesized according to procedures reported by Trnka et al.^5^ The additional NMR spectra and other experimental details for preparing these conjugates are reported in the text or supporting material of Trnka et al.^5^ All solvents and reagents were purchased from Sigma-Aldrich, Acro Organics, Alpha Aesar, or Fisher Scientific and used without purification. The reaction progress was monitored using TLC glass-backed 0.25 mm silica gel 60 plates with fluorescence indicator F264 from EMD Sciences in a solvent system of 5% MeOH in DCM. Compounds were purified using silica gel chromatography with silica gel 60 with a particle size 0.040–0.063 mm, 230–400 mesh ASTM, and a solvent system of 1–9% methanol in dichloromethane. Organic solvents were removed from final products by a rotary evaporator to give hygroscopic pure compounds.

#### 4.1.1 CF_3_-TPP^+^-DC

IUPAC name: decyl tris(4-(trifluoromethyl)phenyl)-phosphonium bromide. Made as previously reported.^5^ Temperature, 140 °C; duration, 72 h. Product obtained as a white solid crust after purification with silica gel chromatography. ^1^H NMR (nuclear magnetic resonance) (300 MHz, CDCl_3_): δ 8.20 (dd, *J* = 12.2, 8.3 Hz, 5H), 7.97 (dd, *J* = 8.3, 2.5 Hz, 5H), 4.32 (m, 2H), 1.61 (m, 4H), 1.20 (m, 12H), 0.83 (t, *J* = 6.8 Hz, 3H). ^19^F NMR (282 MHz, CDCl_3_): δ −63.71 (s, 9F). MS (mass spectrometry) (ESI) *m*/*z*: calculated for (M^+^), 607.2171; found, 607.2202. HPLC (high-performance liquid chromatography) analysis: retention time = 28.4 min.

#### 4.1.2 F-TPP^+^-DC

IUPAC name: decyl tris(4-fluorophenyl) phosphonium bromide. Made as previously reported.^5^ Synthesized by stirring at 120 °C for 24 h to give 4-F-TPP^+^-DC as a white solid crust after purification with silica gel chromatography. ^1^H NMR (300 MHz, CDCl_3_) δ 7.90 (ddd, *J* = 12.9, 8.1, 5.1 Hz, 6H), 7.38 (m, 6H), 3.78 (m, 2H), 1.53 (m, 4H), 1.14 (m, 12H), 0.78 (t, *J* = 6.6 Hz, 3H). ^19^F NMR (282 MHz, CDCl_3_) δ −99.49 (m, 3F). MS (ESI) *m*/*z* calculated for (M^+^): 457.23. Found, 457.12. HPLC analysis: retention time = 26.2 min.

#### 4.1.3 Cl-TPP^+^-DC

IUPAC name: decyl tris(4-chlorophenyl) phosphonium bromide. Made as previously reported.^5^ Synthesized by stirring at 110 °C for 24 h to give 4-Cl-TPP^+^-DC as a white solid crust after purification with silica gel chromatography. ^1^H NMR (300 MHz, CDCl_3_) δ 7.84 (dd, *J* = 12.2, 8.6 Hz, 6H), 7.63 (dd, *J* = 8.6, 2.6 Hz, 6H), 3.84 (m, 2H), 1.51 (m, 4H), 1.14 (m, 12H), 0.78 (t, *J* = 6.8 Hz, 3H). MS (ESI) *m*/*z* calculated for (M^+^): 505.1. Found, 505.3. HPLC analysis: retention time = 28.5 min.

#### 4.1.4 CH_3_-TPP^+^-DC

IUPAC name: decyl tri-4-tolylphosphonium bromide. Made as previously reported.^5^ Synthesized by stirring at 110 °C for 6 h to give 4-CH_3_-TPP^+^-DC as a white solid crust after purification with silica gel chromatography. ^1^H NMR (300 MHz, CDCl_3_) δ 7.49 (dd, *J* = 12.3, 8.3 Hz, 6H), 7.36 (dd, *J* = 8.1, 3.1 Hz, 6H), 3.25 (m, 2H), 2.30 (s, 9H), 1.42 (s, 4H), 1.04 (m, 12H), 0.66 (t, *J* = 6.8 Hz, 3H). MS (ESI) *m*/*z* calculated for (M^+^): 445.3. Found, 445.3. HPLC analysis: retention time = 28.9 min.

#### 4.1.5 OMe-TPP^+^-DC

IUPAC name: decyl tris(4-methoxyphenyl) phosphonium bromide. Made as previously reported.^5^ Synthesized by stirring at 100 °C for 6 h to give 4-OMe-TPP^+^-DC as a white solid crust after purification with silica gel chromatography. Yield, 69%. ^1^H NMR (300 MHz, CDCl_3_) δ 7.60 (dd, *J* = 11.9, 8.8 Hz, 6H), 7.13 (dd, *J* = 8.9, 2.4 Hz, 6H), 3.86 (s, 9H), 3.27 (m, 2H), 1.51 (m, 4H), 1.18 (m, 12H), 0.79 (t, *J* = 6.7 Hz, 3H). MS (ESI) *m*/*z* calculated for (M^+^): 493.2866. Found, 493.2897. HPLC analysis: retention time = 27.7 min.

#### 4.1.6 TPP^+^-DC

Obtained from MedKoo Biosciences as a pure white solid compound. The HPLC retention time is 26.7 min.

### 4.2 Instrumentation and Associated Methods

#### 4.2.1 NMR Spectroscopy

^19^F NMR, ^31^P NMR, and ^1^H NMR were used to monitor degradation or probe xTPP^+^–metal chelator interactions. Experiments were conducted on a 300 or 400 MHz Bruker spectrometer using a 5 mm Bruker liquid probe. ssNake version 1.3 software was used for the analysis of the NMR spectra. Samples (0.7 mL) were prepared in 5 mm NMR tubes with 1 mg/mL of the xTPP^+^-DC conjugate of interest in a solution of at least 50% deuterated cosolvent. The NMR instrument was locked on the deuterated organic cosolvent. All fluorine spectra were generated with 64 scans, on average, phosphorus spectra using 32 scans, and ^1^H measurements with 16 scans.

#### 4.2.2 HPLC

Analytical HPLC was used to monitor product degradation. The system consisted of a Phenomenex Luna C18 100 Å LC (liquid chromatography) column (4.6 mm × 250 mm) and SPD-10A UV–vis detector operated using Shimadzu (Kyoto, Japan). The mobile phases were HPLC-grade water with 0.1% trifluoroacetic acid (TFA) and HPLC-grade acetonitrile (ACN) with 0.1% TFA. The gradient used for analytical HPLC was 10 to 95% ACN/water increasing linearly over 30 min. Semipreparative HPLC was used for hydrolysis product purification. The system consisted of a Phenomenex Luna 5 μm PFP (2) 100 Å LC column (250 × 21.20 mm) and Shimadzu HPLC system using a SPD-20A UV–vis detector. The gradient used on semiprep HPLC was 50 to 95% ACN/water increasing linearly over 30 min.

#### 4.2.3 Mass Spectrometry

Mass spectrometry data for degradation product determination were obtained using a Thermo Q Exactive mass spectrometer utilizing ESI ionization.

### 4.3 Sample Preparation

#### 4.3.1 Stability Studies of xTPP^+^ Conjugates in Buffers and Cosolvent Systems

All stability studies were conducted in a solvent system of 50% organic cosolvent and 50% molecular biology-grade distilled H_2_O or aqueous buffer (1:1 organic:aqueous) with 1 mg/mL of the xTPP^+^ derivative being analyzed. Solutions under these conditions will be referred to as prepared by standard procedures. Tris buffer solutions were prepared with 10 mg/mL Tris free base and HEPES buffer solutions with 23.8 mg/mL HEPES free acid followed by HCl and NaOH, respectively, dropwise until the desired pH was reached. All samples, unless otherwise noted, were stored at 20 °C on a benchtop. Stability studies of CF_3_-TPP^+^-DC in buffer solutions were conducted using ^19^F NMR and analytical HPLC, while other xTPP^+^ derivatives were analyzed using only analytical HPLC.

Ionic strength study solutions were prepared by adding up to 278 mM of either KCl or NaCl to stock solutions of CF_3_-TPP^+^-DC prepared in neutral aqueous DMSO-*d* under standard procedures and analyzed by NMR. The sample of CF_3_-TPP^+^-DC under argon was prepared using the standard solution parameters in aqueous DMSO-*d* at pH 8.3 but in an NMR tube, giving a 0.7 mL solution. The NMR tube was placed with the cap off in a large flask under alternating vacuum and argon gas, three times each, and then removed from the flask, and the NMR tube was sealed. The sample of CF_3_-TPP^+^-DC in the absence of light was prepared by standard procedures in aqueous DMSO-*d* at pH 8.3, but once in an NMR tube for analysis, it was stored in the dark. *N*-Acetyl-l-cysteine studies were conducted by adding 1 mg/mL *N*-acetyl-l-cysteine to a stock solution of CF_3_-TPP^+^-DC prepared under neutral standard conditions with acetonitrile, and analysis was achieved by analytical HPLC. H_2_O_2_ studies were conducted by adding H_2_O_2_ in concentrations of 5 × 10^–9^ M or 0.1 M to standard solutions of xTPP^+^-DC conjugates every 45 min and analyzing with analytical HPLC immediately after adding H_2_O_2_ to the solution.

#### 4.3.2 Studies Evaluating Metal Chelator Interactions with xTPP^+^ Conjugates

Stability studies with DTPA or EDTA present were prepared by adding DTPA or EDTA immediately to the prepared buffer, solvent, and xTPP^+^-DC solution prepared under standard procedures, giving a 1:1 molar ratio of DTPA or EDTA to xTPP^+^ to be analyzed by NMR. Stability studies with CF_3_-TPP^+^-DC and Chelex100 were carried out by either pretreating buffer solution with 50 mg/mL Chelex100 overnight, before filtering Chelex100 and using buffer in standard procedures, or treating a solution containing CF_3_-TPP^+^-DC prepared under standard procedures with Chelex100 and beginning the analysis by NMR and analytical HPLC after 12 h.

NMR studies of metal chelator interactions with TPP^+^-DC or CF_3_-TPP^+^-DC were conducted using Tris and ACN-*d* stock solutions of the xTPP-DC conjugate prepared under standard conditions. The study on titrating DTPA into a solution of TPP^+^-DC was conducted by preparing solutions of (1) 1 mg/mL TPP^+^-DC in 1:1 ACN-*d*:Tris at pH 7.4 with no DTPA and (2) 1 mg/mL TPP^+^-DC in 1:1 ACN-*d*:Tris at pH 7.4 with excess molar equivalent of DTPA. After the analysis of sample 1 by NMR, 100 μL was withdrawn and replaced by solution with excess DTPA, giving a new molar ratio sample of TPP^+^-DC to DTPA, with the same concentration of TPP^+^-DC as the starting solution. This process was continued until a 2:1 DTPA:TPP^+^-DC solution was obtained and analyzed by ^31^P NMR.

## Abbreviations

TPP^+^: triphenylphosphonium
xTPP^+^: triphenylphosphonium having different para substituents on the phenyl rings
OXPHOS: oxidative phosphorylation
ATP: adenosine triphosphate
DMSO: dimethyl sulfoxide
ACN: acetonitrile
CF_3_-TPP^+^-DC: decyl tris(4-trifluoromethyl) phenyl phosphonium
CDCl_3_: deuterated chloroform
EtOD: deuterated ethanol
NMR: nuclear magnetic resonance
HPLC: high-performance liquid chromatography
DTPA: diethylenetriamine pentaacetate
EDTA: ethylenediamine tetraacetic acid
mg/mL: milligrams per milliliter
M: molar
mM: millimolar
